# Water-Deficit Inducible Expression of a Cytokinin Biosynthetic Gene *IPT* Improves Drought Tolerance in Cotton

**DOI:** 10.1371/journal.pone.0064190

**Published:** 2013-05-10

**Authors:** Sundaram Kuppu, Neelam Mishra, Rongbin Hu, Li Sun, Xunlu Zhu, Guoxin Shen, Eduardo Blumwald, Paxton Payton, Hong Zhang

**Affiliations:** 1 Department of Biological Sciences, Texas Tech University, Lubbock, Texas, United States of America; 2 Zhejiang Academy of Agricultural Sciences, Hangzhou, Zhejiang Province, China; 3 Department of Plant Sciences, University of California Davis, Davis, California, United States of America; 4 USDA-ARS Cropping Systems Research Laboratory, Lubbock, Texas, United States of America; University of Delhi South Campus, India

## Abstract

Water-deficit stress is a major environmental factor that limits agricultural productivity worldwide. Recent episodes of extreme drought have severely affected cotton production in the Southwestern USA. There is a pressing need to develop cotton varieties with improved tolerance to water-deficit stress for sustainable production in water-limited regions. One approach to engineer drought tolerance is by delaying drought-induced senescence via up-regulation of cytokinin biosynthesis. The isopentenyltransferase gene (*IPT*) that encodes a rate limiting enzyme in cytokinin biosynthesis, under the control of a water-deficit responsive and maturation specific promoter *P_SARK_* was introduced into cotton and the performance of the *P_SARK_*::*IPT* transgenic cotton plants was analyzed in the greenhouse and growth chamber conditions. The data indicate that *P_SARK_*::*IPT*-transgenic cotton plants displayed delayed senescence under water deficit conditions in the greenhouse. These plants produced more root and shoot biomass, dropped fewer flowers, maintained higher chlorophyll content, and higher photosynthetic rates under reduced irrigation conditions in comparison to wild-type and segregated non-transgenic lines. Furthermore, *P_SARK_*::*IPT*-transgenic cotton plants grown in growth chamber condition also displayed greater drought tolerance. These results indicate that water-deficit induced expression of an isopentenyltransferase gene in cotton could significantly improve drought tolerance.

## Introduction

Water deficit stress is one of the most important factors that affect plant growth and development [1]. Worldwide crop losses due to drought stress have multi-billion dollar impacts to economies annually [2], [3]. Crops grown under rain-fed conditions are the most affected by seasonal variation in rains. Yield integrates many physiological processes that drive plant growth and development and most of these factors are affected by water-deficit stress [4]. Severe drought induced yield reduction has been reported in crops like maize, barley, wheat, rice and cotton [5]–[7]. Water-deficit reduces lint quality and yield in cotton [8]–[10]. Reduction in lint yield in cotton is due to reduced boll production because of fewer flowers and greater boll abortion when stress intensity is greater [7].

Plants have evolved a wide range of molecular programs to sense environmental changes and adapt accordingly to suboptimal growing conditions [11], [12]. Many studies have been conducted to understand the physiological, cellular, and molecular changes in plants in response to drought stress. For example, plants undergo genetic programming for early flowering and accelerated senescence in response to water-deficit stress. Though this has been a natural mechanism for survival under harsh conditions, it has a detrimental effect on productivity and yield in agricultural crops [3]. From an agricultural standpoint, overcoming programmed cell death is a major hurdle in creating drought tolerant crops with minimal yield loss. One approach is to overcome or suppress the drought induced programmed cell death [13]. The other approach is to study the epigenetic mechanisms underlying this early transition from vegetative to reproductive phase under water-deficit conditions [14] and apply this knowledge to avert the early transition to flowering and senescence.

Hormone homeostasis is greatly affected by both biotic and abiotic stresses and the resulting physiological response to stress is closely associated with the levels and balance of hormones [15]. Drought stress typically causes inhibition in synthesis and transport of cytokinins [16]. Recently, Kudoyarova et al. [17] reported that low cytokinin levels were associated with growth inhibition, a decline in stress-tolerance, and onset of senescence. The fact that natural or stress-induced senescence is related to falling levels of cytokinin is well documented [18]. The first evidence of cytokinin delaying leaf senescence dates back to the late 1950 s when Richmond and Lang [19] showed that exogenous application of cytokinin could delay leaf senescence. Subsequently, the molecular basis of cytokinin activity and its role in senescence was established [20]–[22]. Following the discovery of an isopentenyltransferase gene (*IPT*) from *Agrobacterium tumefaciens* and its role in cytokinin biosynthesis [23], efforts were made to express this gene to up-regulate the production of cytokinin to delay senescence [24], [25]. Dexamethasone-inducible overexpression of the *Agrobacterium tumefaciens IPT* leads to higher de novo cytokinin biosynthesis in transgenic Arabidopsis plants [26]. Recent research on the biosynthesis of cytokinin shows that isopentenyltransferase catalyzes a key rate-limiting step in the biosynthesis of cytokinins [26], [27]. Though these early efforts in using *IPT* in transgenic studies were successful in delaying senescence; there were detrimental effects on plant growth and morphology, likely due to altered expression without spatial and temporal regulation of the transgene.

Subsequent efforts were made to regulate the expression of cytokinin biosynthetic genes using the senescence associated gene 12 (*SAG12*) promoter [28]. This strategy involved the developmental targeting of cytokinin biosynthesis in the basal leaves at the onset of senescence and resulted in effective inhibition of leaf senescence. Using this strategy, delayed senescence was achieved in plants like broccoli [29], lettuce [30], rice [31], and wheat [32]. The problem associated with this approach was that *P_SAG12_::IPT*-transgenic plants displayed altered source-sink relationships [33]. Furthermore, *P_SAG12_::IPT*-transgenic plants were also shown to have nutrient deficiencies in young leaves due to inhibition in nutrient remobilization from old to young leaves [30]. Other obvious phenotypes were delayed flowering, reduced seedling establishment under water-limited conditions, and reduced grain fill and yield [34]. This approach was good for plants where leaf or vegetative parts were considered economically important (lettuce, tobacco, cabbage etc.), but not for crops like cereals and millets where grain is the economically important product.

To overcome the problems associated with *P_SAG12_::IPT*, Rivero et al. [35] expressed an Agrobacterial *IPT* (GenBank:X14410.1) [36] under the control of the promoter from a senescence-associated receptor-like kinase gene (*SARK*) from pea [37]. This promoter is a water-deficit and maturation inducible and allowed for regulated expression of *IPT* under maturation and drought conditions [35]. Hence the cytokinin production was not limited to old senescing leaves as in the earlier approach but was also found in all other tissues facing water deficit as well as maturing fruits and grains. Transgenic tobacco displayed a remarkable drought tolerance [35]. Transgenic rice and transgenic peanut harboring the *P_SARK_::IPT* construct displayed delayed response to water-deficit stress with significantly higher yields in comparison to wild-type plants under drought conditions [38], [39]. All *P_SARK_::IPT*-transgenic plants maintained higher water content and higher photosynthetic rates during drought [35].

Upland cotton (*Gossypium hirsutum*) is the No. 1 fiber yielding crop in the world. Creating water-deficit tolerant cotton would benefit cotton production worldwide. To test whether *P_SARK_::IPT* would also confer drought tolerance in cotton, we generated transgenic cotton plants with the *P_SARK_::IPT* construct by using Agrobacterium-mediated gene transfer and analyzed their performance under water-deficit conditions. Here, we report that water-deficit and maturation specific expression of *IPT* in cotton also confers increased drought tolerance.

## Results

### Creation and molecular analysis of *P_SARK_::IPT*-transgenic cotton plants

**Figure 1:**
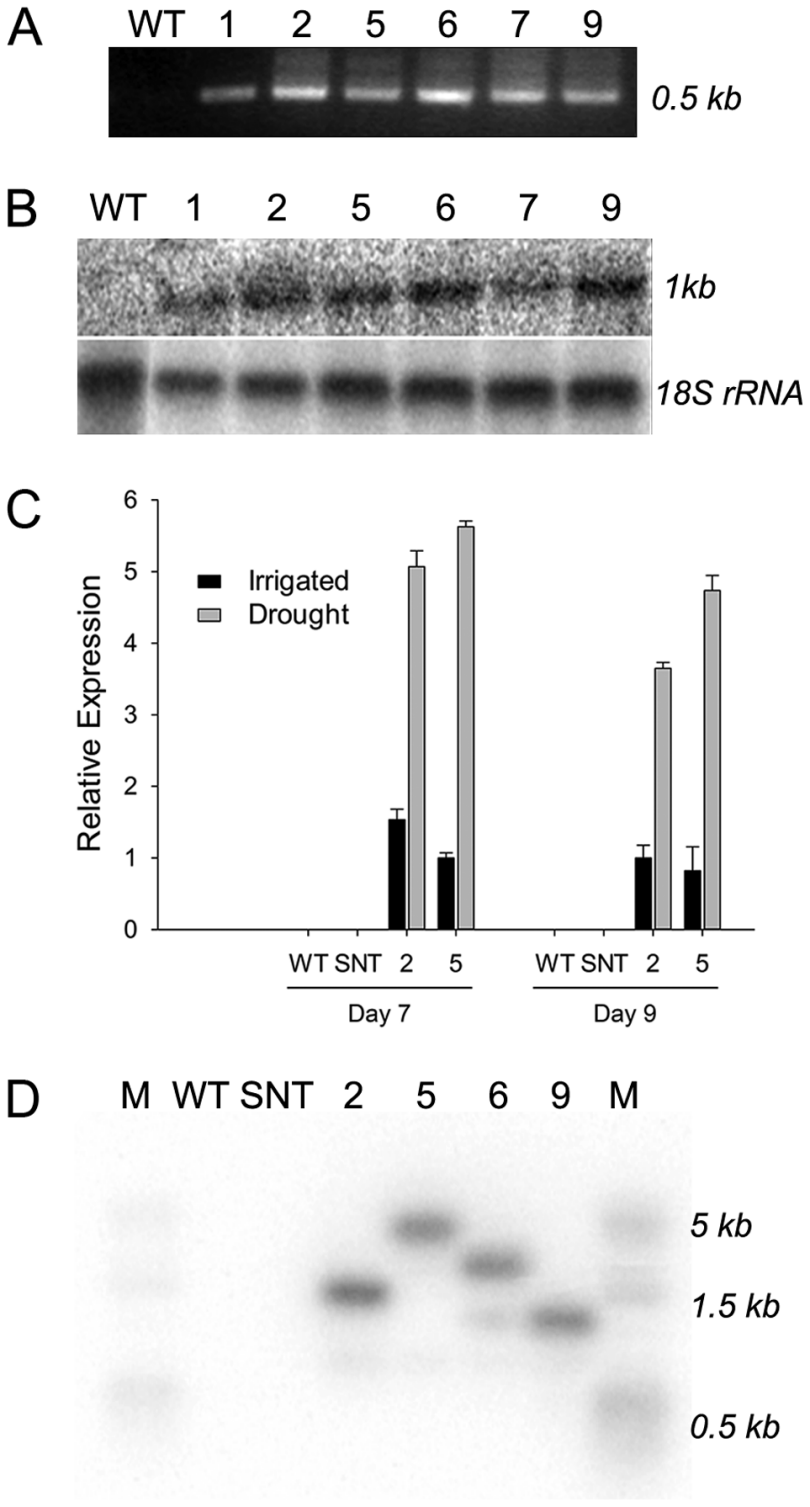
Molecular analysis of *P_SARK_::IPT*-transgenic cotton. **A**. PCR analysis of *P_SARK_::IPT*-transgenic cotton plants using the *SARK* promoter specific forward primer and the *IPT* specific reverse primer. WT, wild-type; 1, 2, 5, 6, 7, and 9, six independent *P_SARK_::IPT*-transgenic cotton plants. **B**. RNA blot analysis of wild-type and *P_SARK_::IPT*-transgenic cotton plants using an *IPT* DNA fragment as a probe. **C**. Relative *IPT* expression in two *P_SARK_::IPT*-transgenic cotton plants under well watered and water-deficit conditions. The quantitative RT-PCR experiments were conducted using the cotton ubiquitin gene *UBQ7* as the internal standard. SNT, segregating non-transgenic. **D**. DNA blot analysis of wild-type, segregating non-transgenic, and four *P_SARK_::IPT*-transgenic cotton plants. M, DNA molecular size marker.

A total of 40 independent transgenic cotton lines carrying the *P_SARK_::IPT* construct were generated through Agrobacterium-mediated transformation. To confirm the integration of the T-DNA from the Agrobacterium Ti plasmid, genomic DNAs were extracted from putative transgenic plants and PCR was performed with two sets of primers. One set involving promoter-specific forward primer and gene-specific reverse primer and the other set involving gene-specific forward and reverse primers were used. An example of a PCR analysis that indicates the presence of the transgene is shown in Fig. 1A. To test if *IPT* is transcribed in these transgenic plants, RNA blot analysis was conducted. Water-deficit stress was imposed on these plants by withholding water for three days before total RNAs were isolated. RNA blot data is shown in Fig. 1B, which indicates that the *IPT* transcript was indeed expressed in transgenic plants after water-deficit treatment. However, the *IPT* expression appeared to be low, because it took a long time (>20 hours) to reveal the hybridizing band (Fig. 1B). We therefore used quantitative real-time (RT)-PCR, a more sensitive technique, to analyze the *IPT* transcript. No *IPT* transcript could be found in wild-type (WT) and segregated non-transgenic (SNT) plants (Fig. 1C), but it was found in well watered *P_SARK_::IPT-*transgenic cotton plants, which is similar to what were reported in *P_SARK_::IPT*-transgenic tobacco plants (35) and *P_SARK_::IPT*-transgenic rice plants (38). Our data confirm that the *SARK* promoter is active under well watered conditions, although at very low levels. However, water deficit did increase *IPT* transcript in *P_SARK_::IPT-*transgenic cotton plants (Fig. 1C). After *P_SARK_::IPT*-transgenic cotton plants were withheld water for 7 days, the *IPT* transcript level was at least 5 folds higher in line 5 and 3 folds higher in line 2 than the *IPT* transcript level under well watered conditions (Fig. 1C). Stable integration of the transgene into the cotton genome was further confirmed with DNA blot analysis by probing the *Eco* RI digested genomic DNA fragments from four PCR positive lines with an *IPT* gene specific probe and the four selected transgenic lines all contained a single copy transgene (Fig. 1D).

### *IPT*-expressing cotton plants display a delayed senescence phenotype

**Figure 2:**
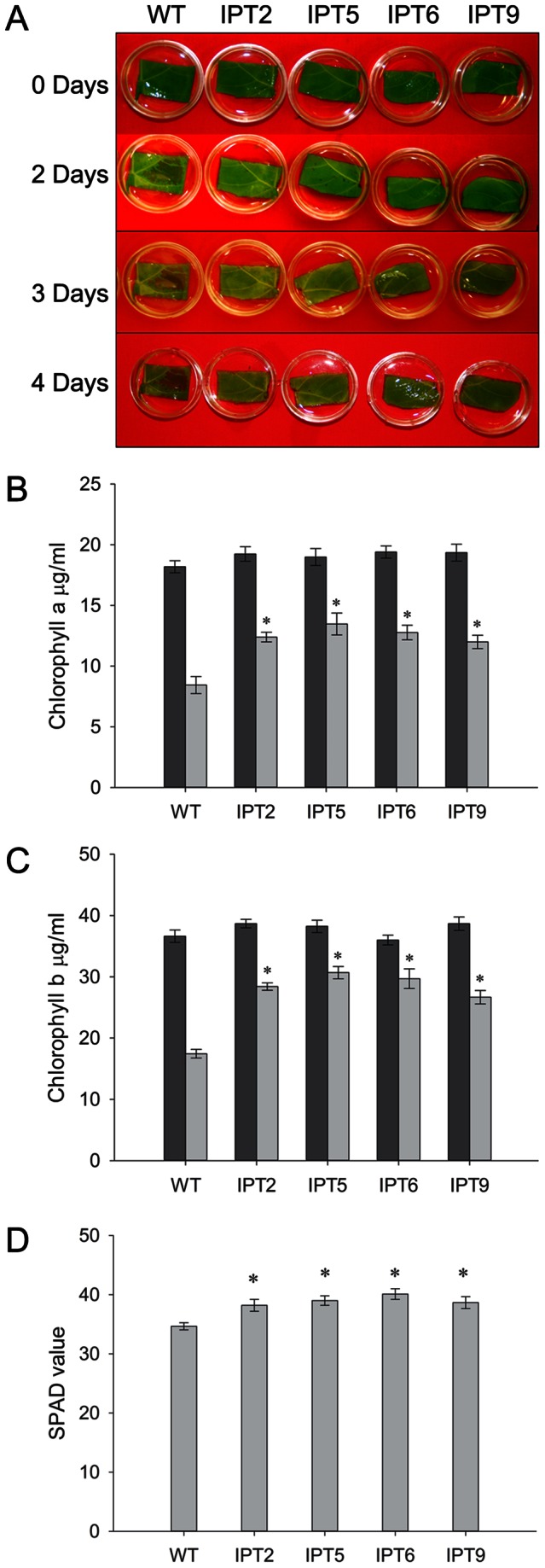
Leaf senescence assay. **A**. Phenotype of detached leaves that were incubated in water for various days in darknessat 30°C. WT, wild-type; IPT2, IPT5, IPT6, and IPT9, four independent *P_SARK_::IPT*-transgenic plants. **B**. Chlorophyll *a* content of wild-type and *P_SARK_::IPT*-transgenic plants before and after 6 days in darkness at 30°C. Dark bar represents chlorophyll content before treatment (0 day) and light bar after treatment for 6 days. Values are mean ± SD (n = 3); * statistically significant at 1%. **C**. Chlorophyll *b* content of wild-type and *P_SARK_::IPT*-transgenic plants before and after 6 days in darkness at 30°C. **D**. Chlorophyll content of wild-type and *P_SARK_::IPT*-transgenic plants assessed by using a SPAD chlorophyll meter. Data were obtained from plants that were under reduced irrigation condition for 60 days in greenhouse and each value was from a total of 15 leaves per plant. Values are mean ± SE (n
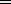
7).

After confirmation of the stable integration of *IPT* into the cotton genome and expression of *IPT* transcript in transgenic cotton plants, we tested whether *IPT* expression in these transgenic lines would delay degradation of chlorophyll in detached leaves. The detached leaves, 3 cm×2 cm in size, from the 3rd fully expanded leaf of WT and four independent transgenic lines, IPT2, IPT5, IPT6, and IPT9, were incubated in water and in darkness for 6 days. There were no significant differences in leaf chlorophyll content in these lines during the initial phase of the treatment (Fig. 2). As the treatment progressed, the chlorophyll content in the leaves of WT plants declined and could be visually scored after 48 h of treatment (Fig. 2A) while transgenic plants remained largely green. By day 6, leaves from *P_SARK_::IPT*-transgenic plants maintained 15% more chlorophyll *a* and 17% more chlorophyll *b* content compared to WT plants (Fig. 2B and 2C). To test whether a similar effect is seen in-planta under drought conditions in greenhouse, we performed the following experiment. In-planta leaf chlorophyll content was measured from 15 leaves, five each from apical, middle, and basal part of the plants using the SPAD chlorophyll meter. There were no significant differences in the leaf chlorophyll content between WT and *P_SARK_::IPT*-transgenic plants under full irrigation conditions (data not shown). As the treatment progressed, the chlorophyll content in the leaves of WT plants decreased in comparing to *P_SARK_::IPT*-transgenic lines. On average, *P_SARK_::IPT*-transgenic plants retained 10–12% higher chlorophyll content than WT plants did after 60 days under reduced irrigation condition (Fig. 2D).

### *IPT*-expressing cotton plants show enhanced tolerance to water-deficit stress under greenhouse and growth chamber conditions

**Figure 3:**
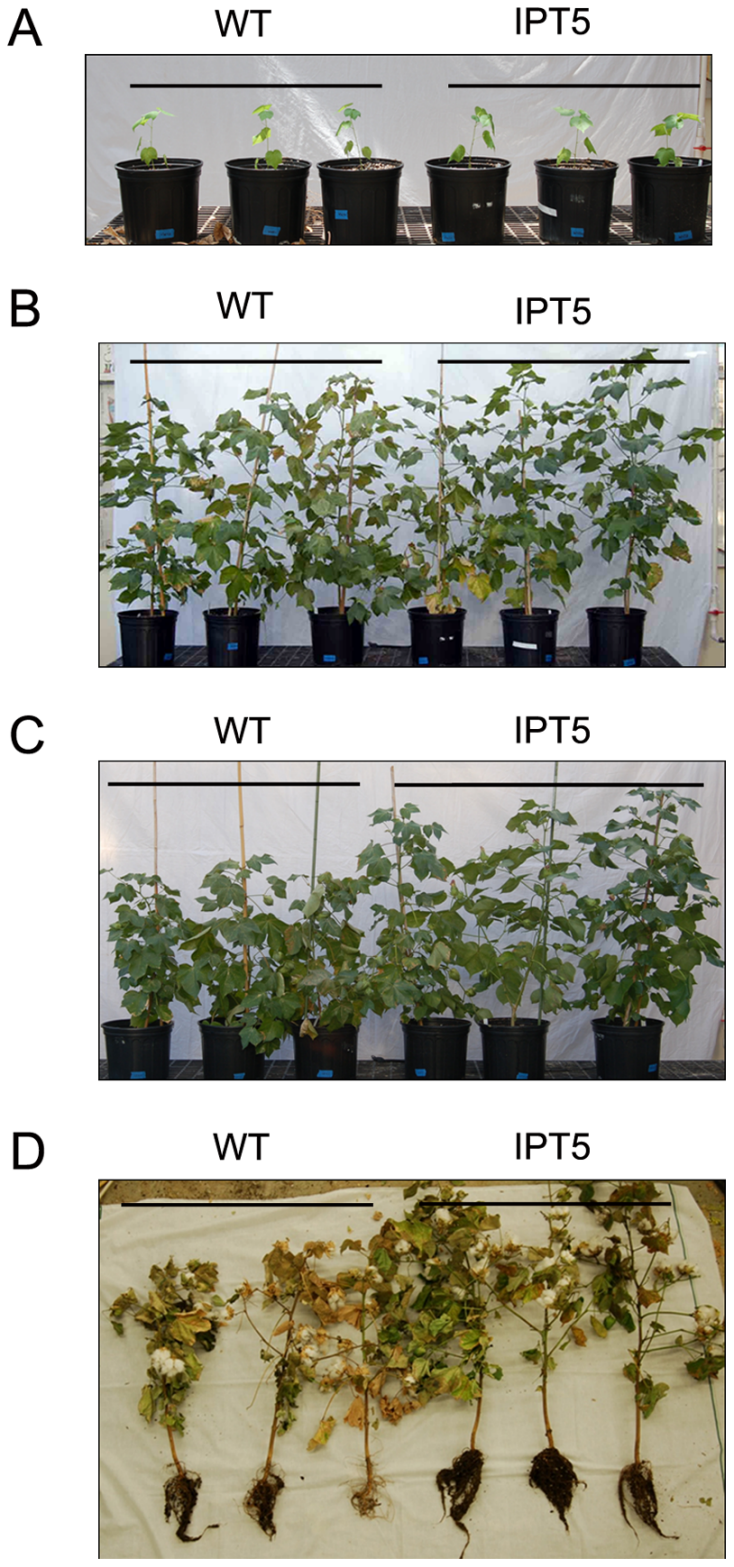
Phenotypes of wild-type and *P_SARK_::IPT*-transgenic cotton plants. **A**. Plants at the beginning of treatment. **B**. Plants after 90 days under regular irrigation condition. **C**. Plants after 90 days under reduced irrigation condition (1/3 of regular irrigation). **D**. Plants at the end of reduced irrigation treatment (120 days).

**Figure 4:**
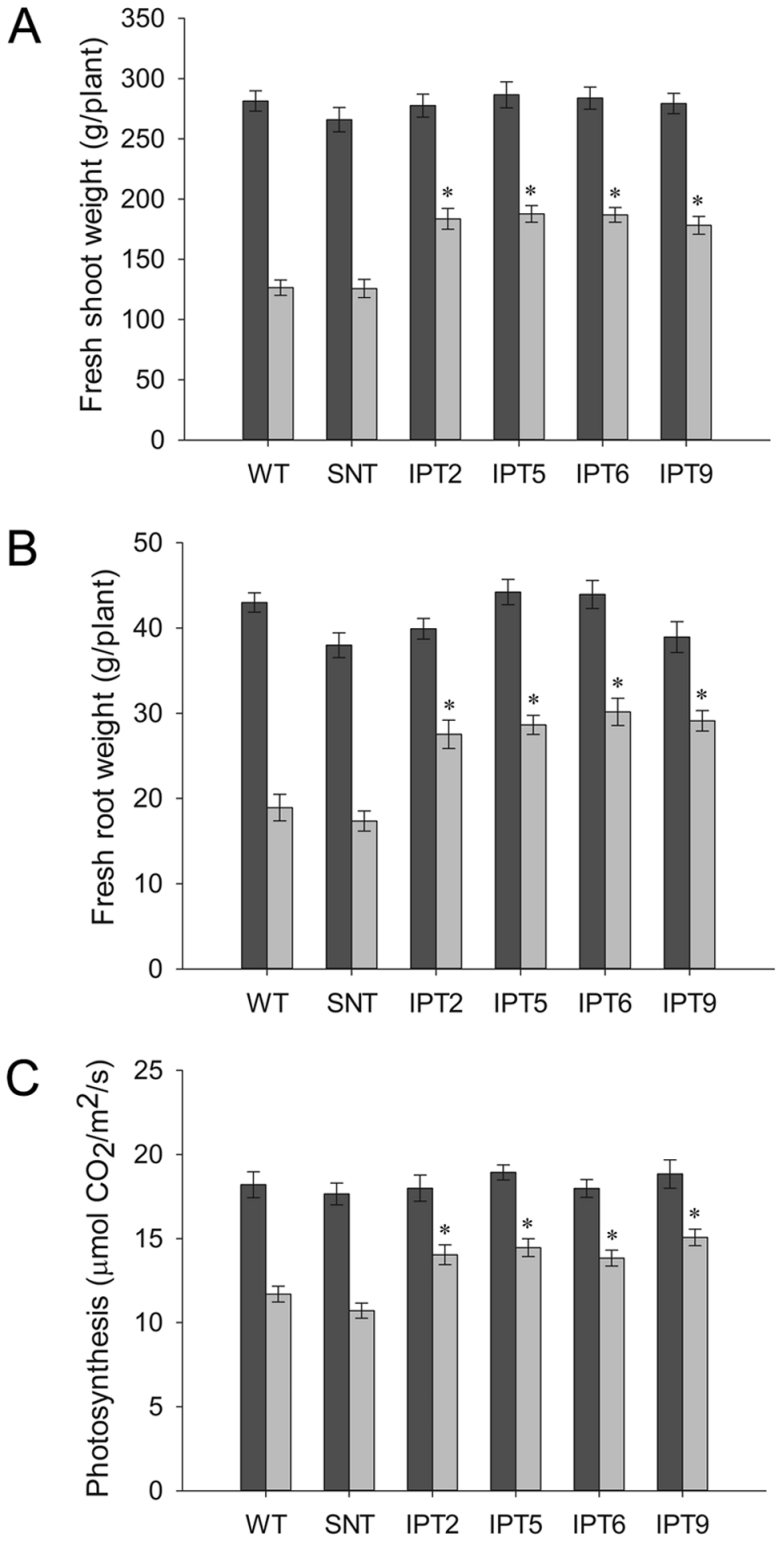
Biomass and photosynthetic analysis of wild-type, segregating non-transgenic, and four independent *P_SARK_::IPT*-transgenic plants under regular irrigation and reduced irrigation conditions in greenhouse. **A**. Fresh shoot weight under regular and reduced irrigation conditions. **B**. Fresh root weight under regular and reduced irrigation conditions. **C**. Photosynthetic analysis under regular and reduced irrigation conditions. WT, wild-type; SNT, segregating non-transgenic; IPT2, IPT5, IPT6, and IPT9, four independent *P_SARK_::IPT*-transgenic cotton lines. Dark bar, regular irrigation; light bar, reduced irrigation. * statistically significant at 1%.

The performance of wild-type plants, segregated non-transgenic plants, and 4 independent *P_SARK_::IPT*-transgenic lines was compared under full-irrigation and water-deficit conditions in the greenhouse. Seven biological replicates were used for each line, and the experiment was repeated three times. Phenotypically, there were no obvious differences between controls and *P_SARK_::IPT*-transgenic cotton plants prior to water deficit treatment or at the completion of study for the fully-irrigated conditions (Fig. 3A and 3B). Under water-deficit conditions, WT and SNT plants showed reductions in overall growth compared to *P_SARK_::IPT*-transgenic plants (Fig. 3C and 3D). There were 50–55% reductions in fresh shoot biomass and 60–65% reductions in fresh root biomass in WT and SNT cotton plants, compared to 35% reductions in fresh shoot biomass and 25% reductions in fresh root biomass in *P_SARK_::IPT*-transgenic plants (Fig. 4A and 4B).

In order to understand the physiology behind the better performance of *P_SARK_::IPT*-transgenic plants under reduced irrigation conditions, photosynthetic performance of the *P_SARK_::IPT*-transgenic plants and control plants grown in greenhouse under well watered and water-deficit conditions were analyzed. Photosynthetic analysis was performed using LiCor 6400 (LI-COR, Inc, Lincoln, NE) and measurements were taken during the recovery phase after re-watering. Under well watered conditions, there were no significant differences in the photosynthetic performance between controls and *P_SARK_::IPT*-transgenic plants (Fig. 4C). Under reduced irrigation conditions, *P_SARK_::IPT*-transgenic plants displayed higher photosynthetic rates (Fig. 4C). Control plants displayed a 30–35% reduction in photosynthetic rates, whereas the reduction was around 20% for *P_SARK_::IPT*-transgenic plants (Fig. 4C).

**Figure 5:**
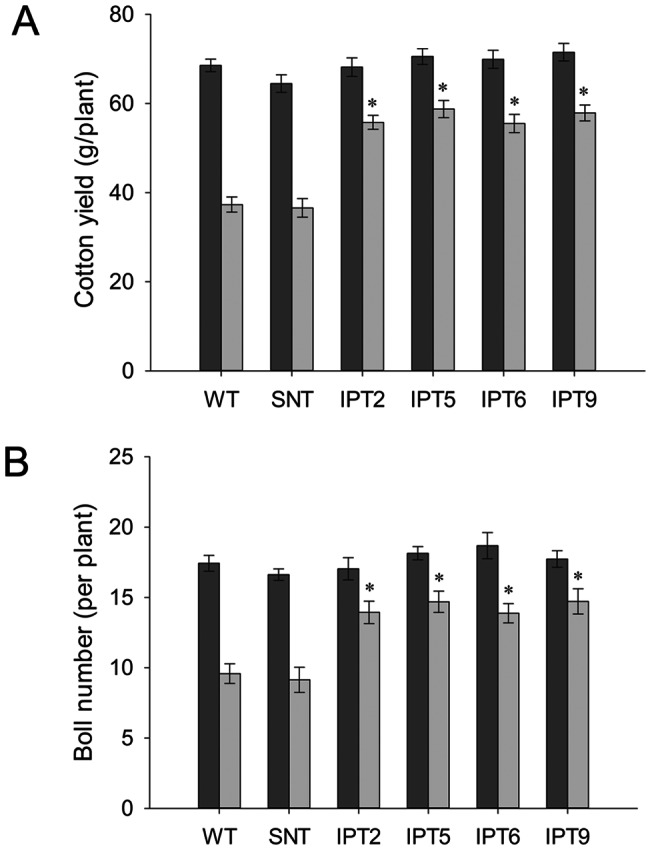
Cotton yield and boll number of wild-type, segregating non-transgenic, and four independent *P_SARK_::IPT*-transgenic cotton plants under regular irrigation and reduced irrigation conditions in greenhouse. **A**. Cotton yield per plant under regular and reduced irrigation conditions. **B**. Boll number per plant under regular and reduced irrigation conditions. Dark bar, regular irrigation; light bar, reduced irrigation. * statistically significant at 1%.

**Figure 6:**
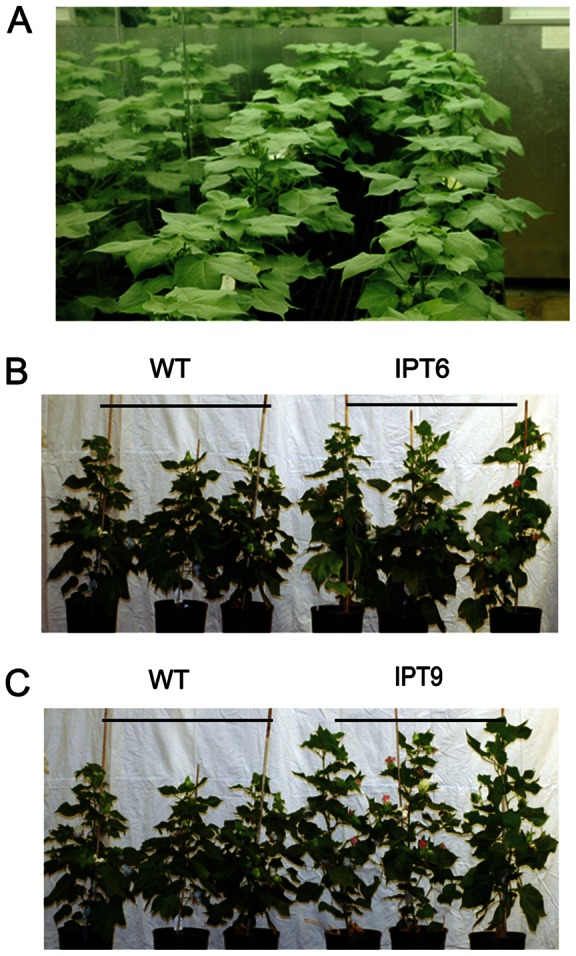
Phenotypes of wild-type and *P_SARK_::IPT*-transgenic cotton plants grown in growth chamber condition. **A**. Plants before treatment. **B** and **C**. Plants after low irrigation treatment for 60 days. WT, wild-type; IPT6 and IPT9, two independent *P_SARK_::IPT*-transgenic lines.

**Figure 7:**
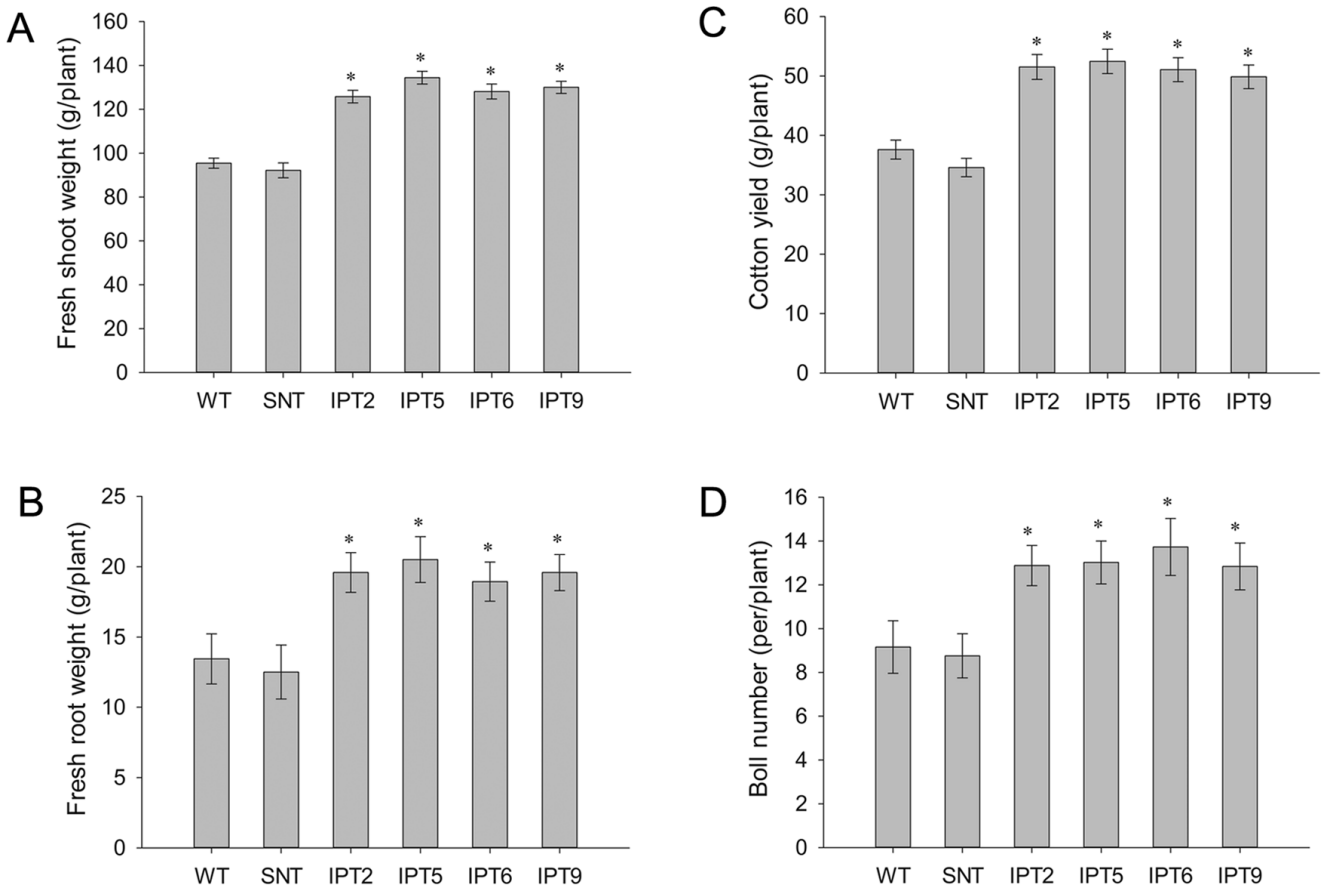
Biomass and yield of wild-type, segregating non-transgenic, and four independent *P_SARK_::IPT*-transgenic plants grown under low irrigation conditions in an Environ growth chamber. **A**. Fresh shoot weight. **B**. Fresh root weight. **C**. Cotton yield per plant. **D**. Boll number per plant. WT, wild-type; SNT, segregating non-transgenic; IPT2, IPT5, IPT6, and IPT9, four independent *P_SARK_::IPT*-transgenic lines. * statistically significant at 1%.

After reduced irrigation treatment, the penalty in terms of the boll number and fiber yield was around 50% of its fully irrigated capacity in control plants, whereas the penalty for *P_SARK_::IPT*-transgenic plants was around 20–25% (Fig. 5A and 5B). In the end, the *P_SARK_*::*IPT*-transgenic lines produced denser and larger root systems in comparison with WT plants under reduced irrigation conditions (Fig. 3D). To test how *P_SARK_::IPT*-transgenic plants would perform in another controlled condition, controls and the four *P_SARK_::IPT*-transgenic plants were grown and drought treated in an Environ growth chamber and five biological replicates were used for each line. Here again, the *P_SARK_::IPT*-transgenic cotton plants outperformed WT and SNT counterparts (Fig. 6). Transgenic plants produced 38% more fresh shoot mass and 51% more fresh root mass than WT and SNT plants did (Fig. 7A and 7B). Overall, transgenic plants produced 30–35% higher yield in comparison with control plants grown under reduced irrigation conditions in the Environ growth chamber (Fig. 7C and 7D). Clearly, the four independent *P_SARK_::IPT*-transgenic lines, IPT2, IPT5, IPT6 and IPT9, displayed significantly improved water-deficit tolerance in laboratory conditions.

### *IPT*-expressing cotton plants display higher photosynthetic capacity and carboxylation rate at saturating CO_2_ than WT and SNT under water-deficit conditions

**Figure 8:**
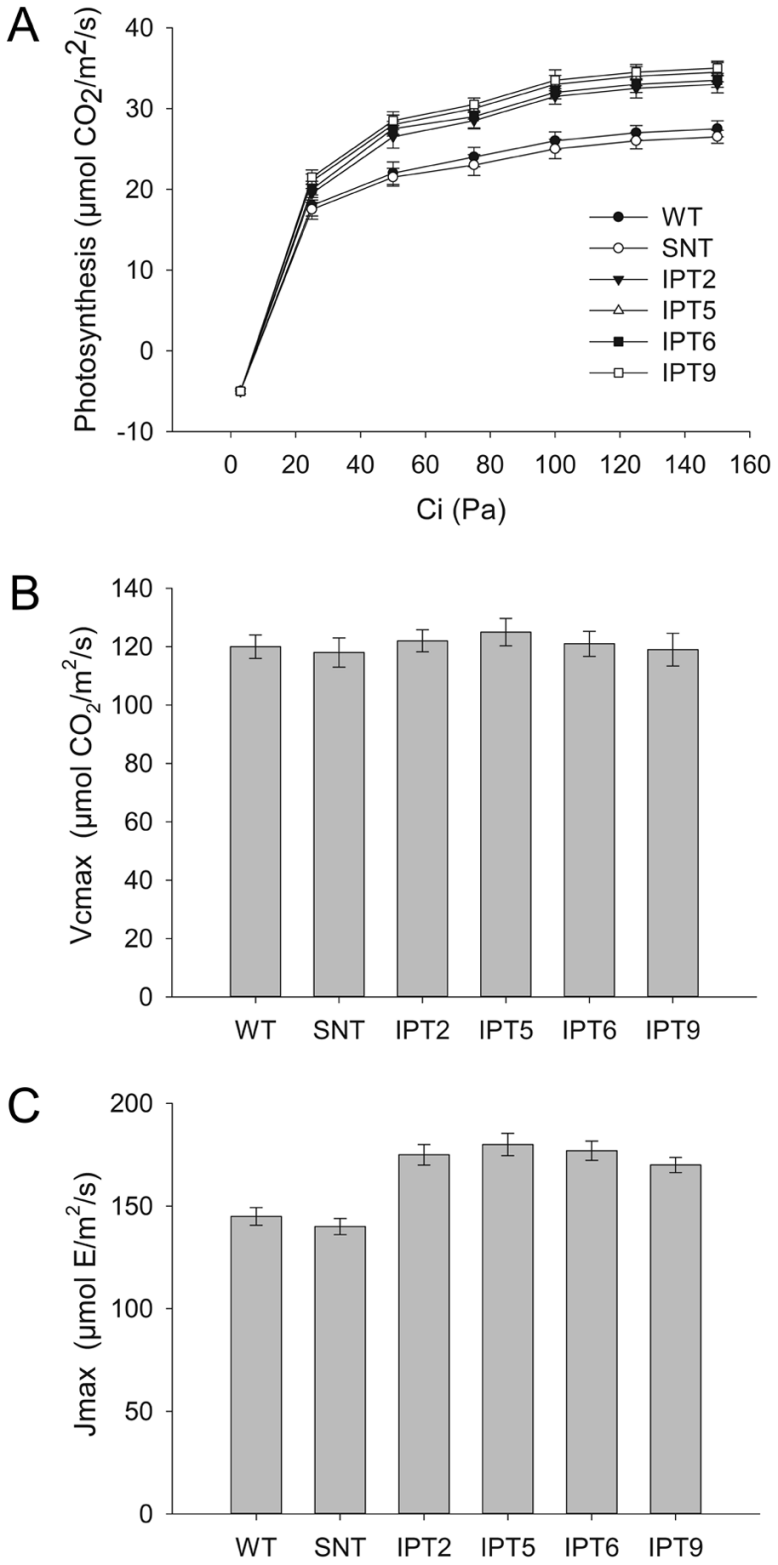
Carbon assimilation rate, Vcmax and Jmax of wild-type, segregating non-transgenic, and four independent *P_SARK_::IPT*-transgenic plants grown under reduced irrigation condition for 60 days. **A**. Carbon assimilation rate (A/Ci curve) at different CO_2_ concentrations. **B**. Estimated maximum rate of carboxylation (Vcmax). **C**. Maximum rate of electron transport (Jmax). Values are mean ± SE (n
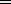
3).

To further study the effect of water-deficit stress on photosynthetic performance, A vs. Ci curves for controls and *P_SARK_::IPT*-transgenic plants were determined under saturating light intensity of 1500 μmol m^−2^ s^−1^, 60 days after drought treatment. Under optimal watering conditions, controls and *P_SARK_::IPT*-transgenic plants displayed similar photosynthetic rates, which increased with a proportional increase in Ci until it reached saturating CO_2_ levels (data not shown). Under reduced irrigation, photosynthetic rates continued to decrease in control plants, whereas it decreased only to some extent in *P_SARK_::IPT*-transgenic plants (Fig. 8A). The data from A/Ci curve were applied to model for photosynthetic response determination using Photosyn software to determine Vcmax and Jmax. There were no significant differences in Vcmax of Rubisco (i.e. ribulose-1,5-bisphosphate carboxylaseoxygenase) in controls and *P_SARK_::IPT*-transgenic plants either under full irrigation or reduced irrigation (Fig. 8B). A significant difference was observed between controls (WT, SNT) and *P_SARK_::IPT*-transgenic plants in the rate of maximum electron transport under reduced irrigation. Jmax was significantly reduced in WT and SNT in comparison with *P_SARK_::IPT*-transgenic plants under reduced irrigation conditions (Fig. 8C). Maximum rate of electron transport, Jmax, is equivalent to the rate of regeneration of ribulose-1,5-bisphosphate (RuBP). Higher rate of Jmax in *P_SARK_::IPT*-transgenic plants might be due to the cytokinin-mediated protection of electron transport under reduced irrigation conditions.

## Discussion

The objective of this study was to create transgenic cotton that would survive severe episodes of drought with minimal yield loss. Here, we demonstrate that regulated expression of *IPT* delays water-deficit induced senescence and enhances drought tolerance. Over the last 20 years, all efforts in using the isopentenyltransferase gene to delay senescence or increase water-deficit tolerance in transgenic plants used the *IPT* gene from *Agrobacterium tumefaciens*, although isopentenyltransferase genes were also found in plants. There are likely three reasons for this phenomenon. Firstly, the Agrobacterium *IPT* gene was cloned almost 30 years ago [23], it was available to scientists much earlier than plant isopentenyltransferase genes. Secondly, although plant isopentenyltransferase genes were identified recently [40], there were few studies on the expression and regulation on these plant isopentenyltransferase genes. It would be risky for plant biotechnologists to use the untested plant isopentenyltransferase genes in creating drought tolerant crops. Thirdly, there were essentially no sequence homology between the Agrobacterium *IPT* and plant isopentenyltransferase genes based on DNA sequence comparisons, it would be better to use the Agrobacterium *IPT* than using plant isopentenyltransferase genes as one would not worry about co-suppression issues caused by similar DNA sequences in transgenic plants.

Plant growth and development in changing environmental conditions are mediated by plant hormones [41]. There is an extensive overlap in the gene expression patterns in plant response to drought and hormone treatment [42]. Plant hormones such as auxin, gibberellins, cytokinins, ethylene, brassinosteroid, jasmonate, abscisic acid (ABA), and salicylic acid have been implicated to have roles in water deficit stress. The level of ABA increases in plants subjected to water-deficit stress. ABA has a two pronged effect in responding to water deficit stress: firstly it helps close the stomata thereby prevent water loss, and secondly it regulates the expression of many genes related to stress response. The reduced photosynthetic rate under water deficit condition is likely caused by limited CO_2_ diffusion due to stomatal closure mediated by ABA [43]. Hormones such as brassinosteroid and jasmonate act concurrently with ABA in promoting senescence and programmed cell death, while hormones like cytokinin, auxin, and ethylene act antagonistically in response to water deficit stress [44]. The levels of cytokinins drop in response to water-deficit [45]. The reduction in the levels of cytokinins is accompanied by the breakdown of proteins and photosynthetic machinery, leading to senescence and programmed cell death.

The major problem with regard to water-deficit stress is an alteration of genetic programming in plants, leading to early flowering and early senescence. This strategy is advantageous to plants for their survival and setting seeds (albeit at reduced levels) or completion of their life cycles, yet it causes a huge yield penalty in annual crop plants. One strategy in improving drought tolerance in plants without compromising much on the yield is overcoming the drought induced senescence. Plant hormone cytokinin has proven to delay senescence in plants: both endogenous up-regulation and exogenous application have been shown to delay senescence in plants. The pioneering work by Gan and Amasino [28] in up-regulating *IPT* under the control of a senescence specific promoter from *SAG12* was successful in delaying senescence in tobacco plants. Since then, this approach has been successfully used in other plants [29]–[32]. However, as indicated in the introduction, *IPT* expression under the control of the *SAG12* promoter led to lower grain yields in transgenic plants [33]. In these plants cytokinin was up-regulated only in the bottom senescing tissues and not in the apical tissues, resulting in a problem with source sink distribution [33]. To overcome the problems associated with the *SAG12* promoter, Rivero et al. [35] expressed *IPT* under the control of the *SARK* promoter (i.e. *P_SARK_*) that came from the senescence associated receptor kinase gene [37]. This promoter is water-deficit and maturation inducible, which is up-regulated during drought and tissue maturation [35]. So with this promoter, the expression of *IPT* was not only up-regulated in the tissues facing water-deficit, but also in the tissues undergoing maturation process. This approach was successful in overcoming the source sink allocation problem because of higher cytokinin levels in the maturing seeds facilitating mobilization of sugars and essential metabolites into them. This approach was successfully demonstrated in both dicot [35], [39] and monocot [38] plants. Here, we demonstrated again that the *SARK* promoter regulated expression of *IPT* could indeed improve drought tolerance in cotton, highlighting the potential that this approach may be applied to other crops.

Photosynthesis is the primary function affected by drought stress [46]. The effect of drought on photosynthesis could be direct or indirect. The direct effect involves decreased CO_2_ availability due to stomatal closure [47]. Indirect effect involves alterations in photosynthetic metabolism [48] as prolonged drought stress can seriously affect the leaf photosynthetic machinery [49]. The effects vary depending on the intensity and duration of drought stress and leaf age [50]. Under prolonged stress, deactivation of the carboxylating enzyme Rubisco has been observed [51]. Other enzymes such as sucrose phosphate synthase, and nitrate reductase have also been shown to display reduced activity [52]. Drought stress leads to alteration in photophosphorylation (i.e. reduced generation of ATP, leading to decreased regeneration of RuBP). An important aspect of prolonged drought stress is the recovery after the stress is alleviated. Recovery after severe water deficit stress is a two-stage process. The first stage involves the acquisition of water by leaves and stomatal reopening [53]. This could be a complete recovery if the stress is for a limited period and water potential does not fall below a sustainable limit. On the other hand, in plants subjected to severe water deficit stress, e.g. a longer time, long enough to damage photosynthetic machinery, photosynthetic recovery is only 40–60% on the day after re-watering and the recovery continues but never reaches its maximum capacity [50], [54]. Bogeat-Triboulot et al. [54] showed that recovery after water stress determined 10 days after re-watering was accompanied by de novo synthesis of photosynthetic proteins such as Rubisco activase and proteins of the water splitting complex. Recent research has shown that impaired photosynthetic biochemistry was the main cause of limited photosynthetic recovery in cotton [55]. For crop yield loss to be minimal, photosynthetic machinery should either be protected from damage or recover quickly after re-watering [56]. Rivero et al. [57] showed that in *P_SARK_::IPT*-tobacco, cytokinin mediated protection of photosynthetic machinery during drought was responsible for better photosynthetic performance during recovery from water-deficit stress.

To study the effect of water-deficit stress on photosynthesis in *P_SARK_::IPT*-transgenic cotton plants, we analyzed the photosynthetic performance of *P_SARK_::IPT*-transgenic cotton plants under normal and reduced irrigation conditions. Transgenic plants and control plants did not display any significant difference in net photosynthetic rates under normal irrigation conditions (Fig. 4C). On the other hand, *P_SARK_::IPT*-transgenic cotton plants grown under reduced irrigation conditions in greenhouse displayed higher photosynthetic rates (Fig. 4C). Analysis of photosynthetic capacity and carboxylation rate at saturating CO_2_ concentration with greenhouse grown plants showed that under reduced irrigation conditions, there was a significant difference in the maximum carboxylation capacity between *P_SARK_::IPT*-transgenic cotton and control plants (Fig. 8A). Even under reduced irrigation conditions, the values of Vcmax remained similar between *P_SARK_::IPT*-cotton and control plants, indicating that higher photosynthetic rates were not due to higher Rubisco activity (Fig. 8B). On the other hand, there was a significant difference in the maximum rate of the electron transport (i.e. Jmax, the rate of RuBP regeneration) between the transgenic lines and control lines (Fig. 8C). Transgenic lines displayed significantly higher Jmax values compared to control lines. Higher rates of RuBP regeneration in *P_SARK_::IPT*-transgenic cotton plants might be the possible reason for higher photosynthetic rates in comparison with control plants (WT and SNT) under reduced irrigation conditions.

In conclusion, based on data from plants grown in our greenhouse and the Environ growth chamber, it is evident that *P_SARK_::IPT*-transgenic cotton plants are indeed more drought tolerant than control plants. *P_SARK_::IPT*-transgenic cotton displayed delayed senescence in response to water-deficit stress and also retained higher chlorophyll content compared to wild-type counterpart. The carbon assimilation in *P_SARK_::IPT*-transgenic cotton was higher, leading to greater biomass and lower yield penalty. Whether the laboratory result will lead to real gains in cotton production in the arid and semiarid areas of the world is not known at this time, but the multi-year and multi-site field trial experiments are undergoing, which should eventually tell if the *IPT* gene can be used for increasing crop production in the water limited areas on this planet.

## Materials and Methods

### Cotton transformation

The *P_SARK_::IPT* construct used in the transformation of three crops, tobacco [35], rice [38], and peanut [39], was used in this study and the cotton variety Coker 312 was used for cotton transformation. The protocol for cotton transformation was mainly from Bayley et al. [58] with minor modifications [59].

### Screening of transgenic lines

T_1_ seeds harvested from T_0_ transgenic lines were delinted with sulfuric acid to remove the lint and washed with water several times to remove traces of acid. The seeds were then surface sterilized with ethanol and bleach as described previously [59], [60]. The seeds were then germinated in tubes containing Stewarts Concentrate supplemented with 50 µg/ml kanamycin. Plants that produced taproot with lateral roots were likely kanamycin positive and transgenic, and plants that produced only taproot without lateral roots were most likely negative or segregating non-transgenic lines (SNT). The presence of *IPT* was then verified by PCR. Screening was continued to the T_4_ generation to obtain homozygous lines. In the process, SNT for each line was obtained. Putative single insertion lines were identified based on segregation ratio and verified later by DNA blot analysis.

### DNA extraction and PCR analysis

Genomic DNAs were extracted from healthy leaves of plants grown under greenhouse conditions by using the method of Guillemaut et al. [61] with minor modifications. About one gram of leaf tissues from young plants were ground in 3 ml of extraction buffer [100 mM NaOAc (pH 4.8), 50 mM EDTA (pH 8.0), 500 mM NaCl, 2% PVP (10,000 MW), 1.4% SDS, and 0.25 mg/ml Ribonuclease A]. The extracts were then incubated at 65°C for 15 minutes to facilitate protein precipitation. They were then centrifuged at 10,000 g for 15 minutes to pellet the debris. The supernatants were carefully transferred into fresh tubes and equal volumes of cold isopropanol were added to facilitate DNA precipitation. The samples were thoroughly mixed by inverting the tubes several times and stored at −20°C for one hour and centrifuged at 10,000 g to precipitate DNA. The precipitates were dissolved in 500 µl of TE buffer (pH 8.0) and extracted with PCI (phenol:chloroform:isoamyl alcohol  = 25∶24∶1). To the aqueous phase, 1/10 the volume of 3 M NaOAc and equal volume of cold isoproponol were added and incubated at −20°C to precipitate DNA. The tubes were then spun at 10,000 g for 15 minutes to pellet the DNA. The pellet was then washed once with 70% ethanol and dissolved in 50 µl of TE buffer (pH 8.0). The DNA was quantified using a Nanodrop equipment(Thermo Scientific, Delaware, USA).

PCR was carried out with a thermalcycler (Mastercycler Gradient, Eppendorf, Hamburg, Germany) using ExTaq DNA polymerase. Two sets of primers were used to confirm the insertion of *P_SARK_::IPT*. One set of primers was designed to amplify a segment of *IPT*. To insure the proper insertion of the promoter, one forward primer was designed in the promoter region. Twenty µl of the PCR reaction mix contained 2 µl of 10× PCR buffer, 50 mM MgCl_2_, 10 mM dNTP mix, forward and reverse primer (10 µM each), Taq DNA polymerase 5 u/µl and 1 µl of DNA template, the remaining volume was made up with water. The PCR was set with an initial denaturation at 95°C for 10 minutes, followed by 32 cycles of denaturation at 95°C for 1 minute, annealing at 55°C for 30 seconds, and extension at 72°C for 1 minute, and a final extension at 72°C for 10 minutes. The oligonucleotide sequences used in PCR are shown below:

P_SARK_-F1: GGTCATTGGGCTTAGGGTTC

IPT-R1: TCGGTTCCTTTCAGTTCTTCC

IPT-F2: CCAACTTGCACAGGAAAGAC

IPT-R2: CTAATACATTCCGAACGGATGAC.

### RNA isolation and RNA blot analysis

About 2 grams of leaf tissues were collected from 3-week old plants grown under greenhouse conditions in potting mix that were subject to water-deficit stress by withholding water for 3 days and ground in liquid nitrogen into fine powders. The powdered tissues were then transferred into a 15 ml tube containing extraction buffer [Tris-HCl 100 mM (pH 9.0), NaCl 200 mM, 1% Sarcosyl, EDTA 20 mM (pH 9.0), and 10% PVP (MW 40,000)], supplemented with 1 ml PCI. The tubes were then centrifuged for 10 minutes at 3,000 g at 4°C. The supernatant was then carefully transferred into a fresh tube and equal volume of 6M LiCl was added. The tube was well mixed by inverting several times and incubated on ice for 1 hour. Then the tube was centrifuged at 3,000 g for10 minutes. The supernatant was transferred into a new tube and 2 ml of 2% potassium acetate was added and well mixed. It was then incubated at 65°C for 5 minutes and centrifuged at 10,000 g for 5 minutes. The supernatant was then transferred to a fresh tube, and 1/10 volume 3M sodium acetate (pH 5.2), and three times volume of ice cold ethanol were added. It was then thoroughly mixed and incubated for 2 hours at −20°C for RNA precipitation. The precipitate was pelleted by centrifugation at 10,000 g for 15 minutes at 4°C. The pellet was then washed twice in 70% ethanol. The pellet was air dried and re-suspended in 20 µl of water. The concentration of RNA was determined using a Nanodrop equipment(Thermo Scientific, Delaware, USA). Samples were used for electrophoresis and RNA blot analysis.

An agarose gel of 1.2% was prepared as described previously [59], [60]. Ten µg of total RNAs were loaded into each well and run for 5 hours. The resolved RNAs from the gel were then blotted onto a Biotrans nylon membrane by capillary transfer overnight. The RNAs were cross linked to the nylon membrane at 1,200 μ J cm^−2^ for 60 seconds in a UV cross linker. The membrane was then air dried at room temperature for two hours. It was then baked at 70°C in a vacuum oven for 1 hour. The membrane was then treated with the pre-hybridization solution [1% BSA, 1 mM EDTA (pH 8.0), 0.5 mM NaHPO_4_ (pH 7.2), 7% SDS] for one hour at 64°C. Gene-specific (*IPT*) and control (18S rRNA) probes were prepared by random priming [62]. The membrane was first hybridized with an *IPT*-specific probe and later with the 18S rRNA probe. Hybridization was carried out at 64°C overnight. After hybridization, the solution was carefully removed, and the membrane was washed once with wash solution I [0.5% BSA, 1 mM EDTA (pH 8.0), 40 mM NaHPO_4_ (pH 7.2), 5% SDS] and twice with wash solution II [0.1 mM EDTA (pH 8.0), 40 mM NaHPO_4_ (pH 7.2), 1% SDS] for 5 minutes each at 64°C. The membrane was then wrapped and exposed to a PhosphorImager screen for 3–5 hours. The PhosphorImager screen was then scanned, and the image was analyzed. The probe bound on membrane was stripped with the stripping solution [2 mM Tris (pH 8.0), 2 mM EDTA (pH 8.0), 0.08% SDS] for 10 minutes at 76°C before it was used for the second hybridization.

### Quantitative real-time PCR analysis

Three-week-old cotton plants, including WT, SNT and *IPT*-transgenic plants, were used for well watered controls and water-deficit treatment. After water was withheld for 7 days and 9 days, one leaf was collected from each plant and put into liquid nitrogen immediately. The total RNAs were extracted by using the Spectrum^TM^ Plant Total RNA Kit (Sigma-Aldrich, St. Louis, MO, USA) according to the manufacturer's instructions. Two µg of total RNAs were used for synthesizing the first-strand cDNA by using the Superscript^TM^ II Reverse Transcriptase of Invitrogen (Carlsbad, California, USA). The oligo(dT)-18 was used as the primer in the reverse transcription reaction. Five µl of diluted cDNA product (20 times dilution from transcription reaction) was used as the template to perform the quantitative RT-PCR analysis with a PCR machine (7500 sequence detection system, Applied Biosystems, Foster city, California, USA) using the Power SYBR® Green PCR Master Mix (Applied Biosystems, Warrington, UK). The final relative expression levels were normalized with the cotton ubiquitin gene *UBQ7* (Genebank No. DQ116441). Three independent biological and three technical replicates were performed. The PCR condition was: 50°C for 2 min, then 95°C for 10 min, followed by 40 cycles of 95°C for 15 sec and 60°C for 1 min.

The primers used are:

IPT-qF1: GCGGGCTTATTCTTGAGGGA

IPT-qR1: TATTCGCCACAAGTTACCCGACCA

UBQ7-qF1: AGAGGTCGAGTCTTCGGACA

UBQ7-qR1: GCTTGATCTTCTTGGGCTTG.

### Genomic DNA extraction and Southern blot analysis

Cotton genomic DNAs from WT, SNT and four independent *P_SARK_::IPT*-transgenic lines 2, 5, 6 and 9 were extracted from the flower bud and the 3rd emerging leaf using Plant Genomic DNA Isolation Kit from MO BIO Laboratories, Inc. (Carlsbad, California, USA). Twenty µg of total DNAs were digested with *Eco* RI and electrophoresed on 0.8% agarose gel, then blotted and hybridized according to the protocol as described in Pasapula et al. [59].

### Detached leaf senescence assay and quantification of chlorophyll *a* and *b*

The third fully expanded leaf from healthy WT and four independent *P_SARK_::IPT-*transgenic lines grown under reduced irrigation conditions in a growth chamber were collected at pre-anthesis stage. Leaves were cut into 3 cm×2 cm rectangles and placed in small Petri dishes containing water. Each sample (WT and transgenic lines) had 3 biological replicates. The leaves were incubated in the dark at 30°C for 6 days. The leaf sections were monitored every 24 hours and the chlorophyll degradation was visually assessed. The changes in leaf phenotype were recorded by photography. Chlorophyll was extracted from 0-day leaf sections and 6-day leaf sections and analyzed by using the methanol extraction method [63].

Leaf sections were incubated in cold methanol for 24 hours at 4°C. The supernatant methanol was poured off into a new container, and 1 ml of this sample was analyzed spectrophotometrically at 652 nm and 665.2 nm to measure the concentration of chlorophyll *a* and *b*. The amount of chlorophyll *a* and *b* were quantified using the following formula based on the molar extinction coefficient of these molecules [63].
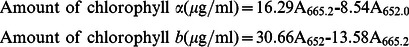



### In-planta chlorophyll measurement using SPAD chlorophyll meter

Chlorophyll content in the leaves of plants grown in the greenhouse was measured using a SPAD chlorophyll meter. Measurements were taken from WT and four independent *IPT* expressing transgenic lines after 60 days under optimal and reduced irrigation conditions. Fifteen measurements were taken per plant; 5 leaves from the top, middle and bottom portion of the plant and the mean value was used for comparison. For each line, readings were taken from 7 plants.

### Drought treatment in greenhouse

WT, SNT and four independent *IPT*-expressing transgenic plants were sown in 3 gallon pots (11.36 L) in potting mixture. Plants were allowed to germinate and establish for a period of 3 weeks before the drought treatments were started. For regular irrigation, 1200 ml of water was added every other day. For reduced irrigation, 400 ml of water was added to each pot every other day. The treatment was continued until boll development and maturation. During the drought treatment, photosynthetic rates were measured. To document the phenotypic differences between controls (WT and SNT) and *IPT*-expressing transgenic lines, pictures were taken. At the end of the treatment, bolls per plant were counted, and fiber yield per plant was analyzed. Fresh root and shoot biomasses were also measured. Seven biological replicates for each line were used. The experiment was repeated three times in greenhouse.

### Drought treatment in growth chamber

WT, SNT and four independent *IPT*-expressing transgenic lines were sown in 2 gallon (7.5 L) pots in potting mix. The plants were allowed to germinate and establish for a period of 3 weeks before the drought treatments were started. For regular irrigation, 900 ml of water was added to each pot every other day; for reduced irrigation, 300 ml of water was added for each pot every other day. The treatment was continued until boll formation and maturation. Photosynthetic rates were measured during the treatment. Pictures were also taken. At the end of the experiment, root biomass and shoot biomass were measured. There were five biological replicates for each line under reduced irrigation. The chamber temperature was set at 30°C, relative humidity was maintained at 60% and photoperiod was set at 16 hours light/8 hours darkness.

### Measurement of leaf gas exchange and photosynthetic rate

To assess the photosynthetic performance of WT, SNT and *IPT*-expressing transgenic lines under normal irrigation and reduced irrigation conditions, gas exchange measurements were taken with a portable photosynthesis system LiCor-6400 (LI-COR Inc., Lincoln, Nebraska, USA). Readings were taken with the 3rd fully expanded leaves of plants that were under full irrigation and reduced irrigation 60 days into the treatment. Environmental parameters in the LiCor measurement chamber were set at temperature 25°C, and air flow rate was set at 500 μmol s^−1^, and light intensity was set at1500 μmol m^−2^ s^−1^. Net photosynthetic rate and transpiration were assessed at a CO_2_ concentration of 400 μmol/mol. The instrument was allowed to warm and stabilize as per manufacturer's instructions. Steady state levels of reference CO_2_ and reference H_2_O were observed before taking measurements. The sample and the reference IRGAs (infra-red gas analyzers) were matched manually before measurements. Five readings were logged for each sample.

### Statistical analysis

Student *t*-test considering one tailed unequal variance was performed to compare the performance of WT, SNT and *IPT*-expressing lines. All *P* values were from comparison between controls (WT and SNT) and transgenic plants. Statistical analysis was performed using Microsoft^®^ Office Excel 2007.
